# Effects of Molecular Distillation on the Chemical Components, Cleaning, and Antibacterial Abilities of Four Different Citrus Oils

**DOI:** 10.3389/fnut.2021.731724

**Published:** 2021-09-01

**Authors:** Feilong Yang, Huijuan Zhang, Guifang Tian, Wenbo Ren, Juan Li, Hang Xiao, Jinkai Zheng

**Affiliations:** ^1^Institute of Food Science and Technology, Chinese Academy of Agricultural Sciences, Beijing, China; ^2^Department of Food Science, University of Massachusetts, Amherst, MA, United States

**Keywords:** citrus oil, components, physicochemical properties, cleaning ability, antimicrobial activity, molecular distillation

## Abstract

Essential oils (EOs) from citrus fruits are excellent aromatic resources that are used in food, cosmetics, perfume, and cleaning products. EOs extracted from four citrus varieties, sweet orange, grapefruit, mandarin, and lemon, were separated into two fractions by molecular distillation. The composition, physicochemical properties, cleaning ability, and antimicrobial activity of each EO were then systematically evaluated. The relationships between each of the aforementioned characteristics are also discussed. In keeping with the principle of “*like dissolves like*,” most citrus EOs show better cleaning ability than acetone and all tend to dissolve the fat-soluble pigment. The key components of citrus EOs are 1-Decanol, α-terpineol, geraniol, and linalool for the inhibition of *Staphylococcus aureus, Escherichia coli, Candida albicans*, and *Vibrio parahaemolyticus*, respectively. The findings of this study will be of significant importance for the effective utilization of citrus peel resources and in the development of future applications for citrus EOs.

**Chemical Compounds Studied in This Article:** (+)-α-Pinene (PubChem CID: 6654); β-Phellandrene (PubChem CID: 11142); 3-Carene (PubChem CID: 26049); β-Myrcene (PubChem CID: 31253); D-Limonene (PubChem CID: 440917); γ-Terpinene (PubChem CID: 7461); Octanal (PubChem CID: 454); Decanal (PubChem CID: 8175); Linalool (PubChem CID: 6549); 1-Octanol (PubChem CID: 957); β-Citral (PubChem CID: 643779); α-Terpineol (PubChem CID: 17100); Hedycaryol (PubChem CID: 5365392); α-Citral (PubChem CID: 638011); 1-Decanol (PubChem CID: 8174); Geraniol (PubChem CID: 637566).

## Introduction

*Citrus*, a genus in the *Rutaceae* family, is an important fruit tree crop widely cultivated in tropical and subtropical regions of the world (1). Most cultivated citrus species are developed by interbreeding the basic taxa (2). Among those species, sweet orange (*Citrus sinensis* L.), grapefruit (*Citrus paradisi* Mac.), mandarin (*Citrus deliciosa* Ten.), and lemon (*Citrus limon* [L.] Burm.) are commercially available worldwide (3). Owing to their pleasant aroma and sweet-sour flavor, citrus fruits are widely consumed as fresh foods or processed into juices, jams, wines, and innumerable other kinds of food (4). The attractive aroma of citrus fruits comes from essential oils (EOs), which are found primarily in the oil sacs or oil glands in the flavedo layer of the citrus peel (5). Citrus EOs are colorless or yellow transparent liquids that are soluble in ether, chloroform, anhydrous ethanol, and petroleum ether, with a density of 0.84–0.87 g/cm^3^ and refractive index of 1.46–1.47. Citrus EOs also have optical rotation because of the rich chiral compositions. These volatile citrus EOs are susceptible to the outside environment as they are sensitive to oxygen, heat, and UV (6). In recent years, citrus EOs have been widely utilized in foods, perfumes, medicine, and cosmetics due to their high yield, attractive aroma, and antimicrobial and antioxidant properties (7, 8).

There are ~200 volatile components in citrus EOs, which can be divided into three major categories: monoterpenes, sesquiterpenes, and their oxygen-containing derivatives (9). Among them, D-limonene is a major component, accounting for 25–97% in the EO of different citrus varieties (10). Although of relatively low content, oxygen-containing mono- and sesquiterpenes are the main aroma-producing components that determine the flavor of citrus EOs (11). Unsaturated straight-chain aldehydes, ranging in length from C_8_ to C_14_, such as citral, citronellal, geraniol, linalool, linalyl acetate, and geranyl acetate, are the primary odiferous components of citrus EOs (12). The physicochemical properties and chemical composition of citrus EOs are affected by their extraction and separation methods. These methods include cold pressing, distillation, and solvent extraction (13). Cold pressing is an economical and effective way to extract citrus EOs with few adverse effects on the quality of the product (14). Molecular distillation is an effective technique for the fractionation of citrus EOs (15–17).

The properties and activities of the volatile components of citrus EOs are determined by their composition and the chemical structures of key components. Many of the volatile components of citrus EOs have proven to be powerful cleaning and degreasing agents. For example, D-limonene can be used in place of noxious organic solvents, such as chlorinated hydrocarbons and n-hexane, as a “green” solvent both for degreasing or for natural products extraction (18, 19). Terpenes in particular are associated with degreasing ability (20, 21). Citrus EOs have also exhibited potent antimicrobial activities. Mandarin EO is shown to inhibit the growth of *Candida albicans, Escherichia coli, Listeria innocua*, and *Staphylococcus aureus*, with inhibition zones ranging from 9.2 to 27.6 mm (22). Terpenes such as γ-terpinene, β-pinene, ρ-cymene, α-terpinolene, and α-thujene in mandarin EO are considered the main contributors to its antimicrobial activity (22). In addition, linalool, citral, geraniol, and decanal play major roles in the activity of citrus EOs against pathogenic microorganisms (7, 23). Citrus EOs also exhibit high fumigant toxicity on insects (24). The characteristics of citrus EOs and their biological activities are affected by the citrus variety, and the extraction and separation methods used to obtain the EOs. However, the relationships among these factors have not been clearly elucidated, limiting the widespread industrial application of citrus EOs.

In this study, crude EOs were extracted by cold pressing from four varieties of citrus fruit, which are sweet orange, grapefruit, mandarin, and lemon. Each oil was then separated into two fractions by molecular distillation. The composition, physicochemical properties, aroma characteristics, cleaning ability, and antimicrobial activity of each fraction were evaluated to reveal any potential relationships among these features. Our findings provide a scientific basis for the practical utilization of citrus EOs in food, perfume, medicine, and other fields.

## Materials and Methods

### Materials

Sweet orange (*C. sinensis* L.), grapefruit (*C. paradisi* Mac.), mandarin (*C. deliciosa* Ten.), and lemon (*C. limon* [L.] Burm.) were grown in Jiangxi province of China and used in this study. High performance liquid chromatography (HPLC) grade ethanol and 6-methyl-5-hepten-2-one were obtained from Fisher Scientific (Shanghai, China). Tween 80 and calcium chloride were purchased from Sinopharm Chemical Reagent Co., Ltd. (Beijing, China). Commercial detergent, corn oil, and machine oil were purchased from a local supermarket in Beijing. Liquid nitrogen was provided by Beijing Shangtong Hong Chemical Co., Ltd. (Beijing, China). Standard strains of *E. coli* (ATCC 43888), *S. aureus* (ATCC 22004), *Vibrio parahaemolyticus* (ATCC 17802), *C. albicans* (CMCC (F) 98001), *Salmonella typhi* (ATCC 14028), and *Pseudomonas aeruginosa* (ATCC 27853) were obtained from Beijing Solarbio Science & Technology Co., Ltd. (Beijing, China). Solid and liquid media for each microbial strain were provided by Beijing Aoboxing Biotechnology Co. Ltd. (Beijing, China). Gentamicin and ketoconazole and phosphate buffer solution (PBS, pH = 7.02, 0.0067 M) were purchased from Sigma-Aldrich (Shanghai, China). Ultrapure water was prepared by a Milli-Q system (Millipore, Bedford, USA).

### Crude Citrus EOs Extraction by Cold Pressing

Crude EOs from four citrus varieties was extracted by the cold press method as described in our previous report with some modifications (25). Citrus peel of each variety (25 kg) was collected from fresh and ripe citrus fruits after cleaning. Then they were soaked in a calcium chloride solution (0.8%) for 5 h at room temperature. Cold pressing of these treated citrus EOs was performed on a Pressofiner (6YL-70, Nanyang Qifeng Machinery Co. Ltd., Henan, China), giving a mixture of citrus EO and water which was immediately centrifuged at 12,000 rpm for 10 min at 4°C (5810R, Eppendorf, USA) to get the separated EO layer. Finally, 500, 480, 455, and 510 ml of EO were obtained from sweet orange, grapefruit, mandarin, and lemon peel, respectively. Afterwards, they were stored in the dark at −20°C before further separation and analysis.

### EO Fractions Preparation by Molecular Distillation

**Figure 1:**
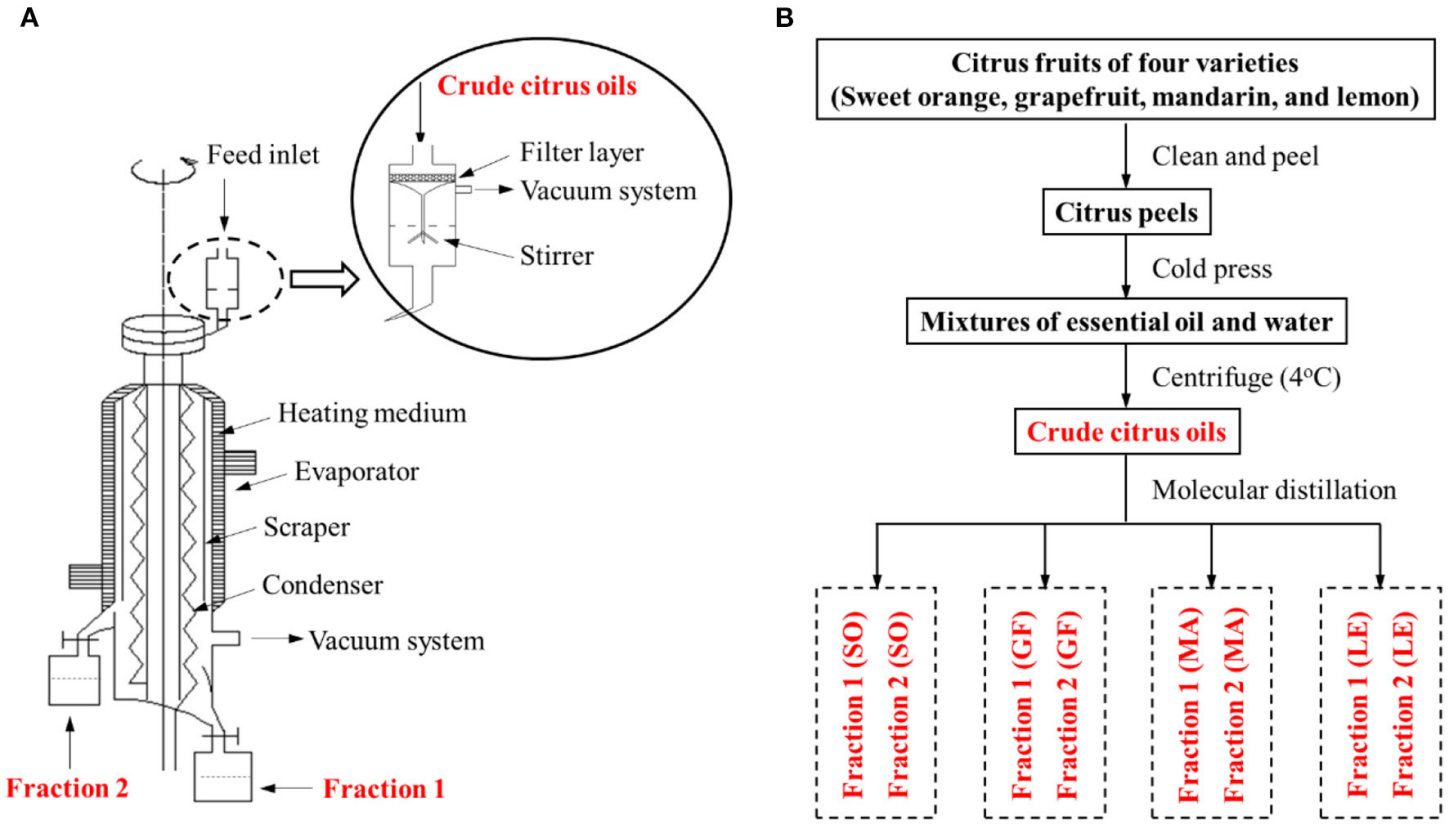
**(A)** Schematic diagram of our molecular distillation apparatus. **(B)** Flow chart of the extraction and separation of citrus essential oils (EOs) from sweet orange (SO), grapefruit (GF), mandarin (MA), and lemon (LE).

Separation of fractions 1 and fractions 2 from citrus crude EOs was performed on a lab-modified wiped-film molecular distillation apparatus as shown in Figure 1A. The citrus crude oils were separated more effectively with filtration, vacuum, and stirring devices configured on our feeding system of molecular distillation. Specifically, 300 ml of the crude EO was fed with the rate at 2.5 ml/min and the operating pressure at 6 × 10^−3^ mbar. The uniformity and unity of the EO film were achieved by setting the film-forming system at 250 rpm of its rotational speed, keeping evaporation temperature and condensation temperature at 65 and 5°C, respectively. Finally, the fractions of citrus EOs were stored in the dark at −20°C before analysis.

### GC-MS Analysis

Analysis of the composition of citrus EO samples was carried through Shimadzu QP 2010 plus gas chromatography coupled with a mass spectrometer detector (GC-MS, Shimadzu Co. Ltd., Kyoto, Japan) which was equipped with DB-WAX capillary column (100 m × 0.25 mm i.d., film thickness, 0.25 μm) and flame ionization detector (FID). Specifically, 1 ml of 30-fold diluted citrus EO samples (dilution with ethanol) and 1 μL of 6-methyl-5-hepten-2-one (99%, internal standard) were mixed in a vial to be delivered to GC-MS. During the analysis process, the GC oven temperature was programmed as that in our previous study (25). Lastly, the retention time of the volatile standards, the Kovats indices, and mass spectra in the NIST 11 databases were all used to identify the components of the four crude EOs and their fractions. The relative content of each component in the citrus EOs was expressed in the form of 6-methyl-5-hepten-2-one equivalents by comparing the concentration-peak area ratios relative to that of the internal standard (6-methyl-5-hepten-2-one).

### Physical Properties

The physical properties, i.e., chromatism, density, viscosity, refractive index, optical rotation, solubility in 90% ethanol, and surface tension, of citrus EO samples were characterized at 20°C according to reported methods with slight modifications (6, 25). Digital Imaging System (Digieye, Verivide, England) was employed to analyze the chromaticity of those samples. Chromatism (Δ*E*) was calculated quantitatively according to the following equation based on the measured L^*^ (luminosity), a^*^ (red–green), and b^*^ (yellow–blue) values of the samples (**Equation 1**).

(1) $$\begin{array}{r} \Delta E=\sqrt{{\left(L^{*}-L_{0}^{*}\right)}^{2}+{\left(a^{*}-a_{0}^{*}\right)}^{2}+{\left(b^{*}-b_{0}^{*}\right)}^{2}} \end{array}$$

Where L^*^, a^*^, and b^*^ belong to the test groups (citrus EOs), while $L_{0}^{*}$, $a_{0}^{*}$, and $b_{0}^{*}\;$ are from the control group (ultrapure water). The density of the citrus EO was measured by the weighing method with an electronic balance (ME204E, Mettler-Toledo, Shanghai, China) involved. The viscosity, refractive index, and surface tension data of the samples were directly measured by a dynamic shear rheometer (Physia MCR 301, Anton Paar, Austria), a digital refractometer (DR102, TO YOU OPTICAL Instrument Co., Shandong, China), and an Attention theta tensiometer (Biolin Scientific, Finland), respectively. Optical rotation determination was achieved by a Polarimeter (341, Perkin Elmer, Shanghai, China). Specific optical rotation ([α]) was calculated as follow (**Equation 2**):

(2) $$\begin{array}{r} \left[\alpha \right]=\frac{\alpha }{l\times \rho } \end{array}$$

Where α represents the optical rotation of the citrus EOs, *l* represents the length of the rotator tube (*d*m), and ρ represents the density value of the citrus EO (g/cm^3^).

### Electronic Nose Analysis

Electronic nose (AIRSENSE Analytics, GmBH, Schwerin, Germany) was used to identify and discriminate aroma characteristics of citrus EO samples by simulating human olfaction (26). Ten gas sensors (W1C, W5S, W3C, W6S, W5C, W1S, W1W, W2S, W2W, and W3S) with a specific signal response for selective components are the key elements of this test instrument (27). The raw data of this electronic sensory apparatus was preprocessed by principal component analysis (PCA) to distinguish the flavor characteristics of the EO samples extracted from different citrus varieties.

### Determination of Cleaning Ability

The cleaning property of the citrus EOs was determined to develop a new type of cleaning agent for industry and household application. A measurement of 2 ml of cooking and machine oil were used to leave representative oil stains on a square white gauze (4 × 4 cm). Additionally, 4 ml of citrus EO samples, acetone, and commercial detergent was used as the agent to clean the stain in the gauze, respectively (only commercial detergent cleaned oil stain with the help of water and a rub). The photos of the degreasing results demonstrate the discrepancy in cleaning effects of the citrus EOs visually.

### Solubility Determination of Pigments With Different Polarities in Citrus EO Samples

The solubility of some common pigments (β-carotene, curcumin, and carmine) in citrus essential oils, n-hexane, acetone, and 95% ethanol were determined. Specifically, these pigments were added to 1 ml of EO samples, n-hexane, and 95% ethanol until they became insoluble. Then the solutions were filtered, 20 μL of which were diluted with dimethyl sulfoxide (DMSO) (200 μL). The absorbance of the dilutions was measured in a 96-well plate under 470 nm (for β-carotene and curcumin) and 512 nm (for carmine), respectively. The essential oils, n-hexane, and 95% ethanol with no pigments, undergoing the same dilution with DMSO, were used as blank controls. The corresponding absorbance serves as a parameter for comparing the ability of the pigments to dissolve in different essential oils, n-hexane, and 95% ethanol.

### The Calculation of Contribution Scores

Based on the principle “*like dissolves like*,” the contribution scores of components were made to roughly explain the degree of their assistance with pigments' solubility in EO samples. First, the optimal log *P* for samples to dissolve each pigment was found as following steps. Found from Scifinder Inc., the log *P* of all the components (shown in Table 1) were used to roughly estimate that of each EOs and control samples; the log *P* of each sample would be equivalent with the sum of products of component percentages and their log *P*. Then plotting the solubility of each pigment in samples on the Y-axis to the ranked log *P* of samples on the X-axis, the relationship between the solubility of pigments and log *P* of samples was found. The figures were analyzed by averaging the solubility values which had insignificant log *P* (*p* > 0.05) and comparing them with other solubility values to find the highest one, whose corresponding log *P* would be the optimal log *P* for the pigment to dissolve in a solvent. Finally, the contribution scores would be calculated as the following equation.

$$\begin{array}{l} \text{Contribution Score}=1-\left|\frac{\log P_{component}-\log P_{optimal}}{\log P_{component}+\log P_{optimal}}\right| \end{array}$$

Log *P*_component_ represented log *P* of each component of EO samples; log *P*_optimal_ represented the relatively optimal log *P* for a solvent to dissolve the corresponding pigment.

**Table 1:** Major components in the citrus essential oils (Eos) determined by gas chromatography with a mass spectrometer (GC-MS).

**No**.	**Rt (min)**	**Components**	**Log** ***P*** **(25°C)**	**Sweet orange oil (mg/L)**			**Grapefruit oil (mg/L)**			**Mandarin oil (mg/L)**			**Lemon oil (mg/L)**		
				**Crude oil**	**Fraction 1**	**Fraction 2**	**Crude oil**	**Fraction 1**	**Fraction 2**	**Crude oil**	**Fraction 1**	**Fraction 2**	**Crude oil**	**Fraction 1**	**Fraction 2**
1	4.20	(+)-α-Pinene	4.321	300	320	254	350	368	222	310	341	228	393	390	340
2	6.88	β-Phellandrene	4.386	432	459	389	523	546	390	623	647	472	550	593	557
3	7.86	3-Carene	4.321	106	118	100	82	86	59	126	130	94	87	96	90
4	8.68	β-Myrcene	4.252	1,093	1,120	979	1,244	1,264	900	1,181	1,263	912	1,294	1,381	1,291
5	10.43	D-Limonene	4.552	19,301	20,258	18,089	22,672	22,292	17,578	20,538	22,168	17,020	22,580	22,698	22,430
6	12.10	γ-Terpinene	4.386	–	–	–	26	26	–	260	251	203	28	29	29
7	14.46	Octanal	4.418	168	166	163	100	62	112	73	65	60	107	92	151
8	26.21	Decanal	3.970	229	191	292	210	74	291	163	101	173	220	154	398
9	29.82	Linalool	2.795	385	359	412	5,856	4,269	6,366	312	256	281	607	558	747
10	30.45	1-Octanol	2.876	–	–	34	248	132	319	104	94	83	255	201	309
11	36.18	β-Citral	3.100	–	–	69	114	–	156	103	55	118	1,860	1,551	2,642
12	37.63	α-Terpineol	2.708	–	–	–	299	96	405	260	124	–	298	214	–
13	37.62	Hedycaryol	4.886	–	–	–	–	–	–	–	–	325	–	–	502
14	39.02	α-Citral	3.127	62	46	90	94	112	136	–	58	135	2,112	1,646	3,207
15	41.73	1-Decanol	3.895	–	–	–	95	–	148	45	–	62	102	45	219
16	46.24	Geraniol	2.942	–	–	–	6,233	1,926	8,239	–	–	–	36	–	90

### Screening of Antimicrobial Activity

The screening of antimicrobial activity of the citrus EO samples was achieved by the disc diffusion test (Kirby–Bauer test) (23, 28, 29). Firstly, 100 μL suspension of the dilute microorganisms (10^6^-10^7^ CFU/mL, diluted with sterile PBS) was seeded into the specific solid medium in Petri dishes (90 mm). Subsequently, a sterile filter paper disk with a diameter of 6 mm was placed onto the surface of the solid medium, soaked with 5 μL of each undiluted citrus EO sample. Gentamicin and ketoconazole were used as positive controls for anti-bacteria and anti-fungi, respectively. The plates were inoculated with strains under their standard culture conditions. Finally, the inhibition zone diameter for each EO sample was measured using a standard millimeter ruler (Deli, Shanghai, China).

### Determination of Minimum Inhibitory Concentration of Antimicrobial Activity

The minimum inhibitory concentration (MIC) of citrus EO samples was measured by the double dilution method with specific liquid culture media in a 96-well plate (30). Using 0.5% tween 80 as emulsifier, the citrus EOs were tested at series of concentrations: 10, 5, 2.5, 1.25, 0.625, 0.313, 0.156, and 0.078% (v/v). Microbial growth was indicated by turbidity readings at 600 nm measured by a microplate reader (Varioskan Flash, Thermo Fisher Scientific, Waltham, MA, USA) after incubation. The lowest concentration of citrus EOs inhibiting microbial growth in the culture medium was considered as their MIC.

### The Calculation of Linear Correlation Coefficient

A linear correlation coefficient was used to indicate the relationship between antimicrobial activity and component content of EO samples. According to antibacterial ability, the EO samples were sorted from weak to strong. Then plotting the percentage of the component in EOs samples on the Y-axis to the corresponding order number (1, 2, 3, …) on the X-axis, the linear correlation coefficient between antimicrobial activity and component was calculated by Excel (Microsoft Inc., USA).

### Statistical Analysis

All individual experimental operations and measurements were performed in triplicates. The data of the results were presented as means ± standard deviation calculations. A one-way analysis of variance (ANOVA) and Duncan's multiple range test (*p* < 0.05) were applied to determine the significance of the difference between the data of different citrus EOs by using SPSS 22 (IBM SPSS Inc., Chicago, IL, USA). Principal component analysis, orthogonal partial least squares discriminant analysis (OPLS-DA) models, and the corresponding contribution scores production were performed on SIMCA 14.1 (Sartorius Stedim Biotech Inc., Malmö, Sweden).

## Results and Discussion

### Chemical Composition of Crude Citrus EOs and Fractions Thereof

**Figure 2:**
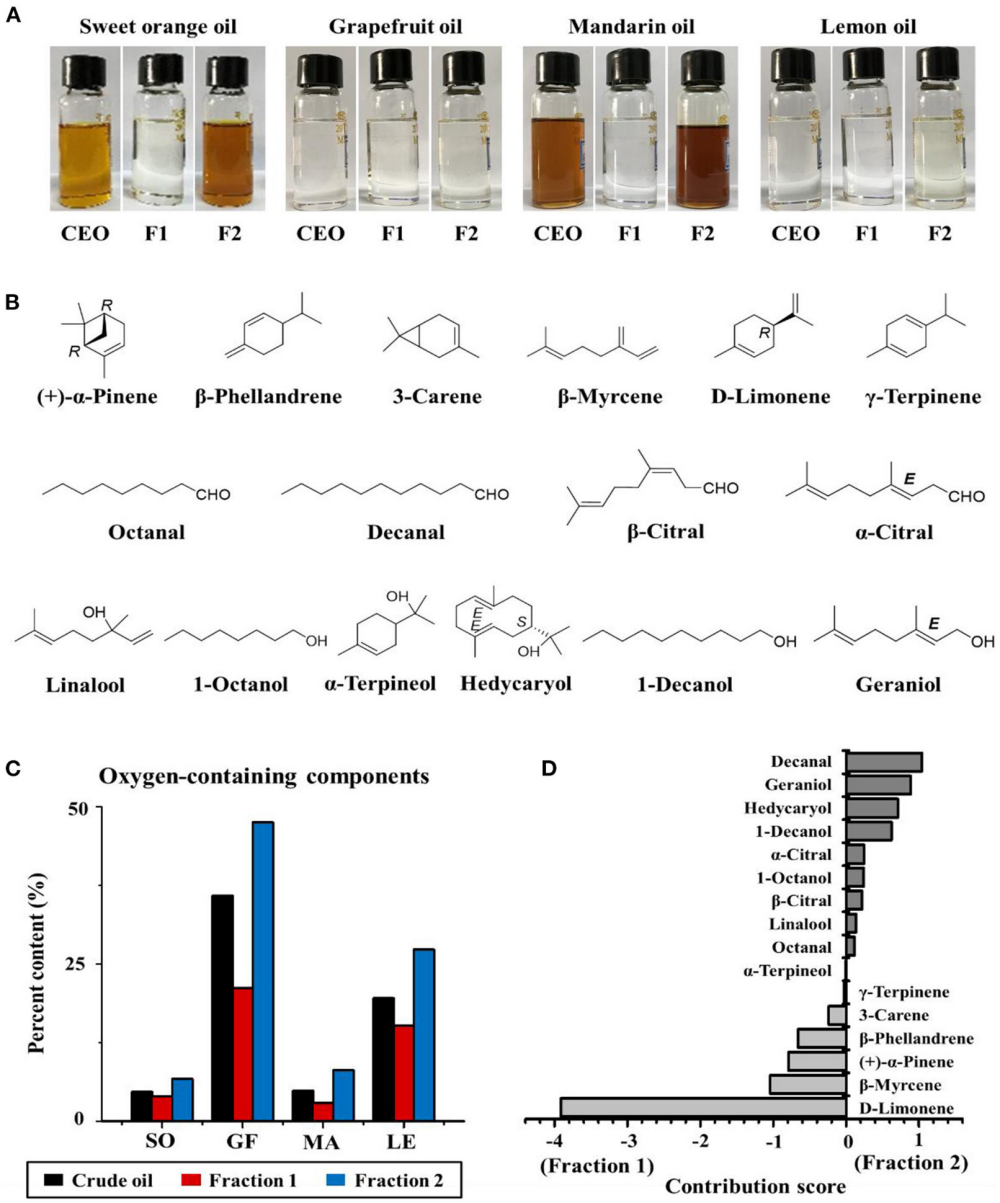
**(A)** The appearance of sweet orange, grapefruit, mandarin, and lemon essential oils (CEO, F1, and F2 represent crude essential oil, Fractions 1 and 2, respectively). **(B)** Chemical structures of the major components of citrus EOs. **(C)** Percentage of oxygen-containing components in crude EOs and Fractions 1 and 2 of sweet orange (SO), grapefruit (GF), mandarin (MA), and lemon (LE). **(D)** Contribution score of each component to the difference between Fractions 1 and 2.

A flow chart describing the extraction and separation of EOs from sweet orange, grapefruit, mandarin, and lemon is shown in Figure 1B. Each crude citrus EO was divided into two fractions using a lab-modified molecular distillation apparatus. Colorless, low molecular weight volatiles with a longer free path reached the condenser plate while heated under vacuum. These collected in the distillate stream were named Fraction 1. High molecular weight components with shorter free paths returned to the heating plate, resulting in an enriched residue stream, which was named Fraction 2 (Figure 2A). The chemical compositions of the crude EOs and their fractions were determined using GC-MS with full dissociation and identified by matching GC peak retention times, Kovats indices, and the MS spectra of the detected compounds with corresponding standards. The results are presented in Table 1. A total of 37 components were identified and divided into seven subcategories: monoterpenes, sesquiterpenes, aldehydes, esters, alcohols, ethers, and phenols. Sixteen major volatiles, accounting for over 99% of the total detected essential oil components, were identified as (+)-α-pinene, β-phellandrene, 3-carene, β-myrcene, D-limonene, γ-terpinene, octanal, decanal, linalool, 1-octanol, β-citral, α-terpineol, hedycaryol, α-citral, 1-decanol, and geraniol. The chemical structures of the 16 components are shown in Figure 2B. Note that the first six volatiles to elute were terpenes, followed by oxygen-containing components primarily consisting of fatty aldehydes and alcohols. Although the composition of volatile components in the investigated EOs varied, D-limonene (49.78–87.94%, with a concentration over 17,020 mg/L, shown in Table 1) was the most abundant component of all the crude EOs and their fractions, in agreement with previous reports (31).

**Figure 3:**
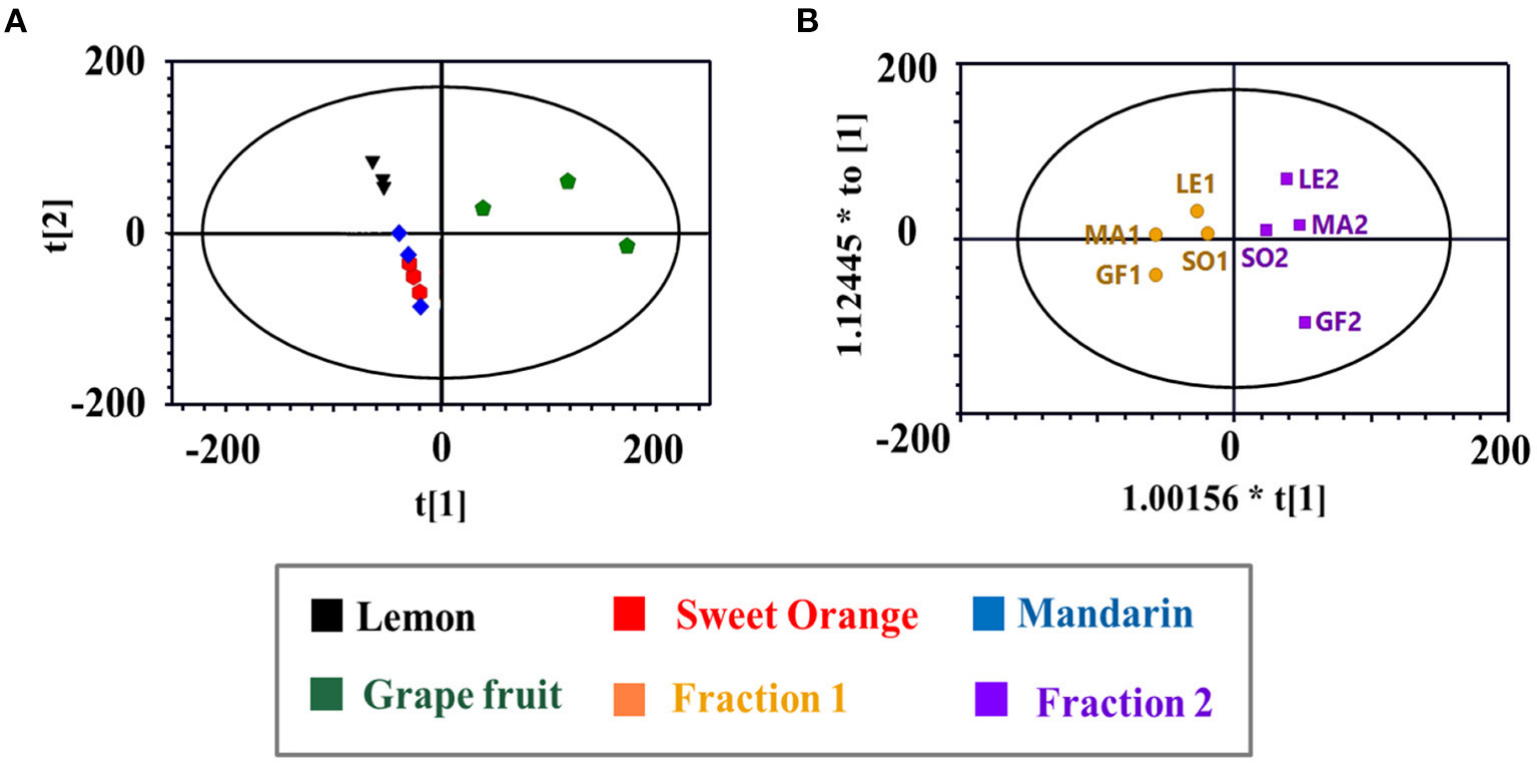
**(A)** The principal component analysis (PCA) scores plot of citrus EOs samples; **(B)** The orthogonal partial least squares discriminant analysis (OPLS-DA) scores plot of the two fractions of citrus EOs samples. Each point represented an individual EOs sample. Compositions were labeled in the loading plot.

Principal component analysis and OPLS-DA models were applied to the GC-MS data to reveal content discrepancies between samples. The chemical compositions of mandarin and sweet orange were similar, but lemon and grapefruit each exhibited unique chemical compositions (Figure 3A). More than 93% of the sweet orange and mandarin EOs were terpenes, including D-limonene, β-myrcene, and β-phellandrene, whereas grapefruit and lemon EOs contained significantly more oxygen-containing components at 35.87 and 19.15%, respectively (Figure 2C). These results indicate that citrus variety strongly affects the composition and concentration of volatile components in citrus EOs. Figure 3B shows the significant differences between Fraction 1 and Fraction 2. Note that D-limonene contributed most of the content discrepancy, although other discrepancies were also evident. Furthermore, components with a higher content in Fraction 1 were denoted with negative values, while those with a higher content in fraction 2 are denoted with positive values (Figure 2D). Generally, the highest amounts of oxygen-containing components were found in Fraction 2 samples, followed by crude EOs, and Fraction 1 samples. These results suggest that the effective separation of Fraction 1 and Fraction 2 from the citrus crude EOs was achieved by the separation method and the relatively large free path components were enriched in Fraction 2.

### Differences in Physical Properties and Aroma Profiles Between Citrus EO Samples

**Figure 4:**
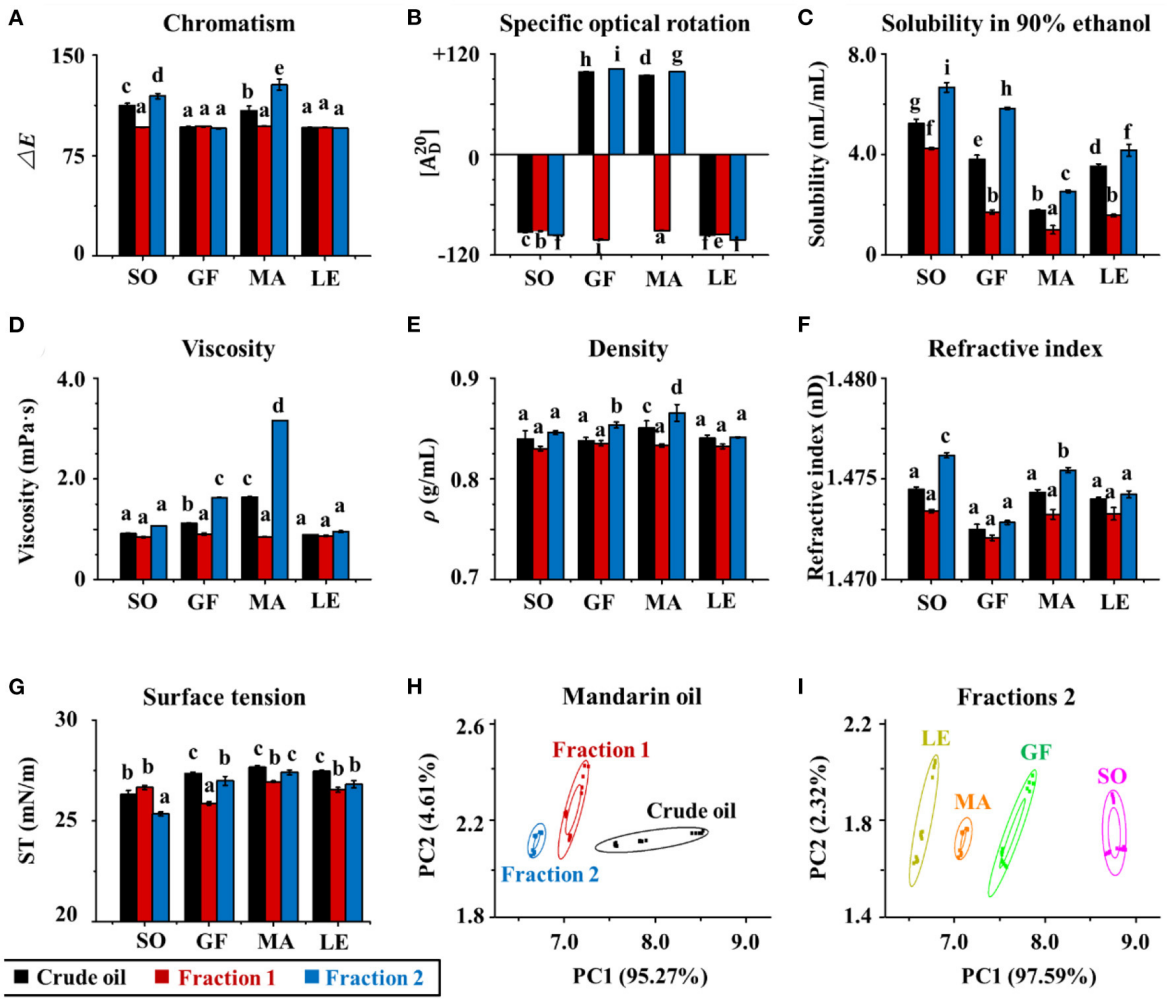
**(A)** Color, specific, **(B)** optical rotation, **(C)** solubility in 90% ethanol, **(D)** viscosity, **(E)** density, **(F)** refractive index, and **(G)** surface tension of crude essential oils (CEOs) and Fractions 1 (F1) and 2 (F2) of SO, GF, MA, and LE. **(H)** Electronic nose analyses (PCA) of crude EOs and Fractions 1 and 2 of MA oil. **(I)** Electronic nose analyses of Fractions 2 of SO, GF, MA, and LE.

Physical properties of each citrus EO were characterized, including color, optical rotation, solubility in 90% ethanol, viscosity, density, refractive index, surface tension, and the aroma profile (Figures 4A–I). Color differences between the EOs were quantified in terms of **Δ*E*** (Figure 4A). In general, the **Δ*E*** of sweet orange and mandarin oils were significantly higher than those of grapefruit and lemon oil crude oils (*p* < 0.05), except for Fraction 1. This indicated that the former contained more natural pigments, such as carotenoids (5, 32). The total carotenoid content of mandarin EO was the highest, followed by those of orange and grapefruit. In contrast, all fractions of grapefruit and lemon EOs and Fraction 1 samples of sweet orange and mandarin oils were colorless. The order of Δ*E* for sweet orange and mandarin oils was Fraction 2 > crude EO > Fraction 1. The above results indicated that all Fraction 1 samples and lemon and grapefruit oils contained smaller amounts of natural pigments, whereas the crude oils and Fraction 2 samples of sweet orange and mandarin contained greater amounts of colored substances. The color of citrus essential oils was related to natural pigments that were enriched in Fraction 2 following molecular distillation.

The solubility of each EO and fraction in 90% ethanol was also evaluated (Figure 4C). The solubility of different subgroups differed significantly, with Fraction 2 > crude EOs > Fraction 1. This may be because Fraction 2 contained more oxygen-containing compounds (mainly alcohols and aldehydes) with polarities similar to that of ethanol. In contrast, Fraction 1 contained more hydrocarbon compounds such as D-Limonene. These results were consistent with the “*like dissolves like*” concept. The trend in viscosity was similar to that of solubility, but no differences were observed between the viscosities of sweet orange and lemon oils (Figure 4D). The result reflected differences in composition among the three subgroups, i.e., Fraction 1, Fraction 2, and crude oils, because the viscosity of a mixture is often determined by the viscosity of its contents. Comparing the density, surface tension, and refractive index of the three subgroups, all the data followed a similar trend that was Fraction 2 > crude EOs > Fraction 1 (shown in Figures 4E–G). Overall, this series of experiments showed that the physical properties of EOs were affected by the composition. Density, viscosity, refractive power, surface tension, and solubility in 90% ethanol increased with higher concentrations of oxygenated species such as geraniol and decanal.

The aroma of citrus EOs is often used to create a romantic atmosphere, alleviate stress, and enhance communication (33, 34). Here, the characteristic aroma profiles of citrus EOs were determined using an electronic nose. The raw data were collected by 10 sensors (W1C, W5S, W3C, W6S, W5C, W1S, W1W, W2S, W2W, and W3S) and further analyzed using PCA in WinMuster software (Version 1.6.2, Airsense Analytics Inc., Germany). Original variables were obtained by classifying the principal components of citrus EOs. The odor characteristics of crude EOs and their fractions were shown in Figures 4H,I. The cumulative variance contributions of principal component 1 (PC1) and principal component 2 (PC2) were more than 99.9%, which showed that the aroma profiles of both crude EOs and their fractions, and for each of the four citrus varieties, were easily differentiated by PCA. Data points corresponding to each oil and fraction clustered in separate regions, indicating significant differences in the odors of EOs of different citrus varieties. These data also showed that EOs were well-separated by molecular distillation. Mandarin EO was tested as a representative sample. Furthermore, comparisons between EOs and fractions from the four citrus varieties show clear differences in aroma, particularly in Fraction 2. Taken together with component analyses, which showed enriched levels of geraniol, decanal, 1-decanol, hedycaryol, 1-octanol, octanal, citral, and linalool in Fraction 2, it can be deduced that these components were primarily responsible for the unique aroma of each EO. The particularly strong responses of sensors W1S, W5S, and W2W also suggested that odor differences were strongly influenced by levels of ethanol, oxynitride, and aromatic and organic sulfur-containing compounds, as well as some oxygen-containing volatiles like linalool and geraniol (35).

### Cleaning Abilities of Citrus EO and Common Detergents

**Figure 5:**
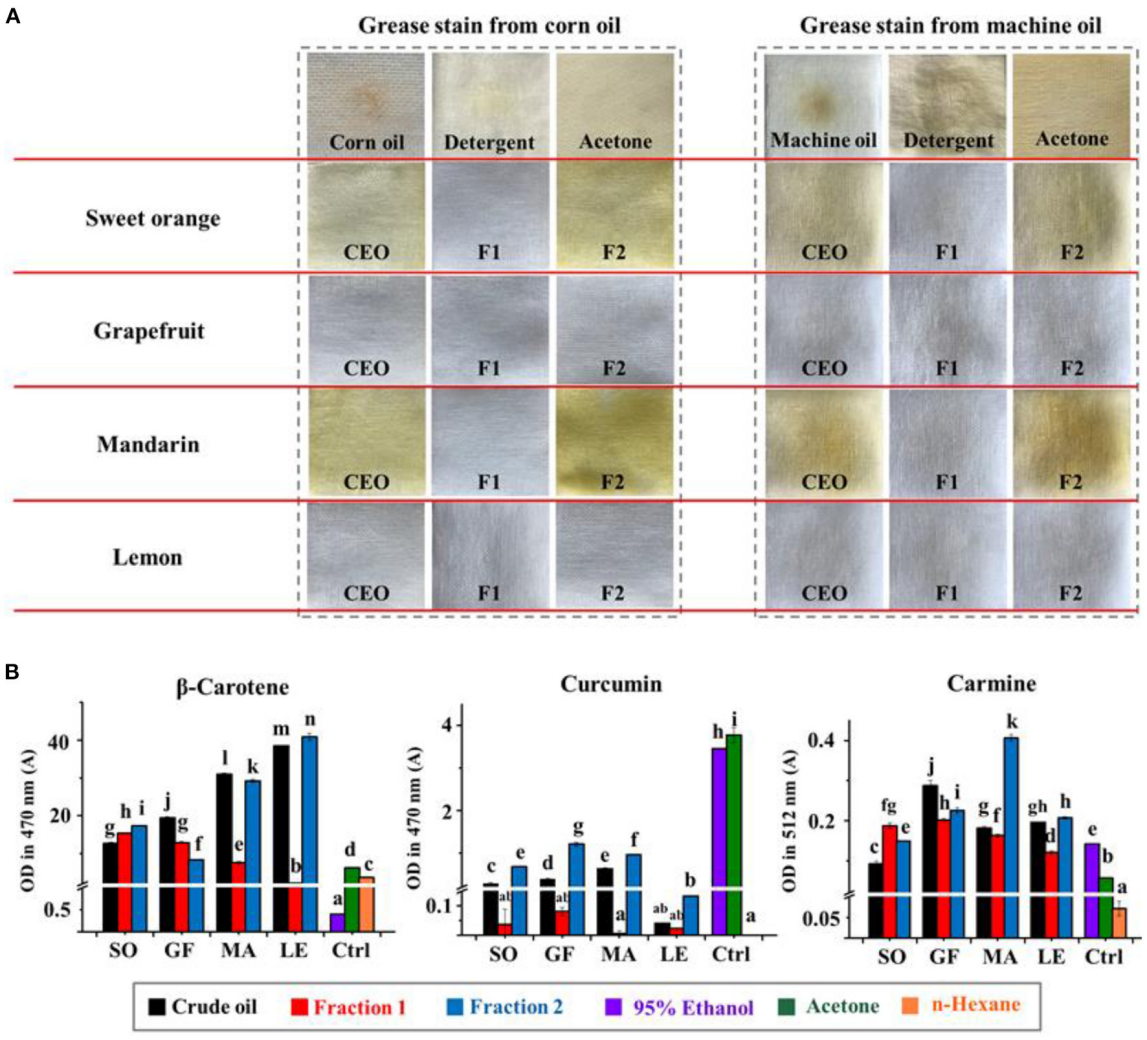
**(A)** The degreasing abilities (corn oil and machine oil) of crude essential oils (CEOs) and Fractions 1 (F1) and 2 (F2) of SO, GF, MA, and LE. **(B)** The solubility of curcumin, β-carotene, and carmine in citrus EO samples. Ctrl indicates the control group, including 95% ethanol, acetone, and n-hexane.

The cleaning and degreasing properties of each EO and fraction thereof were evaluated against commercial corn oil and machine oil. The data in Figure 5A showed that our EO samples were more effective than detergent and acetone in removing grease. Comparisons between different EO samples showed that the colorless oils, i.e., all the Fraction 1 samples and lemon and mandarin oils, exhibited better degreasing abilities than crude EOs and Fraction 2 samples of sweet orange and mandarin, which were yellow or dark yellow. The order of degreasing ability for citrus EOs was opposite to that of Δ*E*. In addition, non-volatile substances, such as pigments in the EOs, can be retained in the material being cleaned. The real cleaning and degreasing effects of citrus EOs may be masked by the color of these pigments to some extent. The data in Table 1 shows that the high level of terpenes and low polarity of aldehydes and alcohols, especially D-limonene with a 49.78–87.94% share, were closely related to the cleaning ability of a given citrus EO (20, 21).

We then evaluated the solubility of common pigments in citrus EOs and traditional organic cleaning agents like 95% ethanol or acetone to assess their relative cleaning abilities. Higher solubility for a given pigment would suggest that it could be more easily removed from a piece of fabric or substrate. The results in Figure 5B show that, compared with 95% ethanol and acetone, citrus EOs had better solubility for β-carotene, the pigment which has less polarity. For curcumin with relatively higher polarity, Fraction 1 appeared low solubility to it while Fraction 2 can have the most dissolved. All EO samples had little solubility for hydrophilic carmine. These results conformed to the trend of EO samples to dissolve low polarity substances (oil stain). A solvent with low polarity, namely n-hexane, has shown a solubility trend to dissolve more fat-compatible β-carotene and the least of water-soluble carmine while 95% ethanol as well as acetone, the medium polar solvents, dissolved the most of curcumin with medium polarity, the empirical rule “like dissolves like” seems feasible in this test. Therefore, based on this principle and the order of solubility for pigments of the EO samples, a kind of index was designed to suggest the degree of contribution of the solvents to dissolving pigments (Table 2). Generally, hydrocarbons [(+)-α-pinene, phellandrene, carene, myrcene, limonene, terpinene, octanal, and decanal] contributed more to the dissolving ability of EOs than oxygen-containing components (linalool, 1-octanol, β-citral, α-terpineol, hedycaryol, α-citral, 1-decanol, and geraniol). This is especially when the polarity of solutes gets lower, as inferred from the comparison between β-carotene and curcumin. For carmine, all citrus EO samples have shown little dissolving ability to it, hence the contribution scores of their components were around zero. Together with the content of low polar components in citrus EOs, the above results can prove that it was low polar components that gave citrus EOs the ability to clean oil stains, especially highly contained D-limonene.

**Table 2:** Contribution scores for the cleaning ability of pigments and linear correlation coefficients between the antimicrobial activity and citrus oil components.

**Component**	Cleaning ability (contribution scores)^*^			Antimicrobial activity (correlation coefficients)^**^			
	**β-carotene**	**Curcumin**	**Carmine**	***S. aureus***	***E. coli***	***V. parahaemolyticus***	***C. albicans***
(+)-α-Pinene	0.9872	0.9572	−0.0196	–	–	–	–
Phellandrene	0.9798	0.9497	−0.0193	–	–	–	–
Carene	0.9872	0.9572	−0.0196	–	–	–	–
Myrcene	0.9953	0.9652	−0.0200	–	–	–	–
Limonene	0.9612	0.9312	−0.0186	–	–	–	–
Terpinene	0.9798	0.9497	−0.0193	–	–	–	–
Octanal	0.9761	0.9461	−0.0192	0.3292	0.1121	–	0.0382
Decanal	0.9704	0.9995	−0.0214	0.6151 (s)	0.3257	0.0019	0.2279
Linalool	0.7978	0.8268	−0.0305	0.0652	0.1933	0.6318 (s)	0.3664
1-Octanol	0.8115	0.8407	−0.0296	0.6719 (s)	0.4386	0.3206	0.2692
β-Citral	0.8479	0.8774	−0.0275	0.0048	–	0.0495	–
α-Terpineol	0.7827	0.8115	−0.0315	0.0339	0.5611 (s)	–	0.6386 (s)
Hedycaryol	0.9259	0.8961	−0.0173	0.1543	0.0171	–	0.0171
α-Citral	0.8522	0.8817	−0.0272	0.0056	–	0.0588	0.0945
1-Decanol	0.9609	0.9910	−0.0218	0.6837 (s)	0.4011	0.1833	0.3158
Geraniol	0.8225	0.8518	−0.0290	0.3065	0.5279 (s)	0.524 (s)	0.6287 (s)

* *The higher contribution scores the components have, the greater they promote pigments to dissolve in EO samples*.

** *s (strongly relative, r^2^ > 0.5); m (medium relative, 0.2 < r^2^ < 0.5). For Antimicrobial activity, only positive correlations are flagged*.

### The Antimicrobial Activities of Citrus EOs

**Figure 6:**
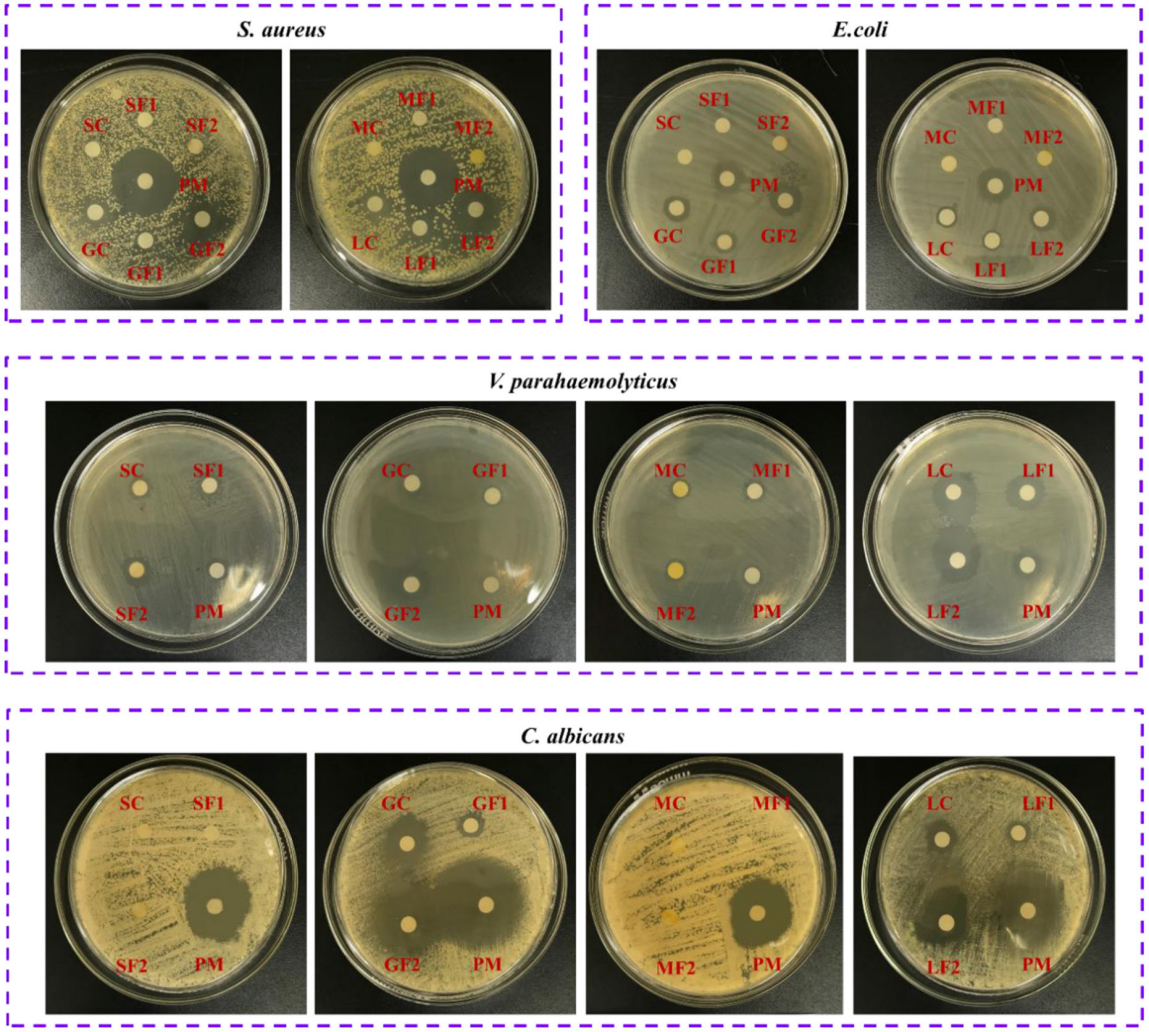
The inhibition effects of citrus EOs against *S. aureus, E. coli, V. parrahaemolyticus*, and *C. albicans* (SOC, sweet orange crude oil; SOF1, fraction 1 of sweet orange oil; SOF2, fraction 2 of sweet orange oil; GFC, grapefruit crude oil; GFF1, fraction 1 of grapefruit oil; GFF2, fraction 2 of grapefruit oil; MAC, mandarin crude oil; MAF1, fraction 1 of mandarin oil; MAF2, fraction 2 of mandarin oil; LEC, lemon crude oil; LEF1, fraction 1 of lemon oil; LEF2, fraction 2 of lemon oil; PM, positive medicine).

Preliminary screening of the antimicrobial activities of undiluted citrus EOs was performed using disc diffusion assays with filter paper discs 0.6 cm in diameter (Figure 6). Significant differences (*p* < 0.05) were observed among the 12 citrus EO samples in their antimicrobial activities against a gram-positive bacterium (*S. aureus* ATCC 22004), gram-negative bacteria (*E. coli* ATCC 43888, *V. parahaemolyticus* ATCC 17802, *S*. *typhi* ATCC 14028, and *P. aeruginosa* ATCC 27853), and fungi (*C. albicans* CMCC [F] 98001). The data in Table 3 shows that grapefruit and lemon oils had inhibitory effects on four kinds of microbes, namely *S. aureus, E. coli, V. parahaemolyticus*, and *C. albicans*, whereas mandarin and sweet orange oils only inhibited *V. parahaemolyticus*. None of the tested oils inhibited *S*. *typhi* or *P. aeruginosa*. These results suggest that the antimicrobial activity of citrus EOs is strain-dependent and not determined by the Gram characteristics of the bacteria. Grapefruit oil had the strongest inhibitory effects against those bacteria except *V. parahaemolyticus*. For *V. parahaemolyticus*, the order of inhibition ability was lemon oil > grapefruit oil > mandarin oil > sweet orange oil. In addition, Fraction 2 samples consistently outperformed Fraction 1 samples and crude EOs in terms of antimicrobial activity. Not surprisingly, the minimum inhibitory concentration (MIC) results were generally consistent with those of disc diffusion assays. However, MIC analyses indicated that grapefruit oil was more effective against *V. parahaemolyticus* than lemon oil, whereas sweet orange oil exhibited the lowest antimicrobial activity.

**Table 3:** The inhibition zone diameters and minimum inhibitory concentrations of citrus EOs against *Staphylococcus aureus, Escherichia coli, Vibrio parahaemolyticus*, and *Candida albicans*.

**Citrus oil**		**The inhibition zone diameters (cm)**				**The minimum inhibitory concentration (%, v/v)**			
		***S. aureus***	***E. coli***	***V. parahaemolyticus***	***C. albicans***	***S. aureus***	***E. coli***	***V. parahaemolyticus***	***C. albicans***
Sweet orange oil	Crude oil	–	–	0.83 ± 0.11*b*	–	–	–	0.313	–
	Fraction 1	–	–	0.73 ± 0.04*ab*	–	–	–	0.313	–
	Fraction 2	–	–	0.93 ± 0.25*bc*	–	–	–	0.156	–
Grapefruit oil	Crude oil	1.40 ± 0.10*c*	1.13 ± 0.13*c*	1.13 ± 0.04*c*	1.90 ± 0.14*d*	2.5	0.625	0.156	0.625
	Fraction 1	0.82 ± 0.08*b*	0.75 ± 0.05*b*	0.93 ± 0.04*bc*	0.95 ± 0.07*bc*	>10	10	0.156	>10
	Fraction 2	1.73 ± 0.25*d*	1.93 ± 0.31*d*	1.30 ± 0.07*c*	2.65 ± 0.21*e*	5	0.313	0.156	0.156
Mandarin oil	Crude oil	–	–	1.05 ± 0.21*c*	–	–	–	1.25	–
	Fraction 1	–	–	0.93 ± 0.04*bc*	–	–	–	1.25	–
	Fraction 2	–	–	1.10 ± 0.14*c*	–	–	–	1.25	–
Lemon oil	Crude oil	1.17 ± 0.12*c*	0.77 ± 0.03*b*	1.75 ± 0.07*d*	1.25 ± 0.07*c*	5	2.5	0.156	>10
	Fraction 1	0.97 ± 0.06*bc*	0.67 ± 0.03*a*	1.50 ± 0.14*c*	0.90 ± 0.14*bc*	5	5	0.313	>10
	Fraction 2	1.63 ± 0.15*d*	0.85 ± 0.09*b*	1.95 ± 0.07*d*	1.65 ± 0.21*d*	5	2.5	0.156	>10
0.1 mg/mL positive medicine (Gentamicin or ketoconazole)	2.75 ±0.22*e*	1.11 ± 0.07*c*	0.99 ± 0.09*bc*	2.95 ± 0.44*e*					

*'a, b, c, d' denote statistically difference. The data with the same letter means they are not statistically different*.

The data in Table 2 further demonstrates the relationship between EO composition and antimicrobial activity. For *S. aureus*, a strong correlation with the inhibition effect was shown by 1-octanol (0.6719), 1-decanol (0.6837), and decanal (0.6151). As sweet orange and mandarin oils also contained a similar content of decanal but processed no inhibition effect on *S. aureus*, decanal were obviously not the contributor to the inhibition of EOs on *S. aureus*. Furthermore, few inhibition effects of decanal on *S. aureus* are also reported (36). On the other hand, 1-octanol has been reported to inhibit *E. coli* instead of *S. aureus* (37, 38), which also excluded 1-octanol from the contributor list. Thus, grapefruit and lemon oils inhibited *S. aureus* mainly under the influence of 1-decanol. Considering the reported excellent inhibition ability of geraniol and α-terpineol on *E. coli* (39, 40), geraniol and α-terpineol with a strong correlation (0.5279 and 0.5611, respectively) may be the decisive factor for grapefruit and lemon essential oils to inhibit *E. coli*. Likewise, geraniol and α-terpineol with strong correlation (0.6287 and 0.6386, respectively) may be the main contributor to the inhibition effect of grapefruit and lemon EO samples on *C. albicans* while linalool (0.6318) and geraniol (0.524) may be the key factor for all the citrus EO samples to inhibit *V. parahaemolyticus*. As for some compounds which linked their content and antibacterial activity with a medium correlation, such as geraniol for *S. aureus* (0.3065), 1-octanol and 1-dectanol for *E. coli* (0.4386 and 0.4011), 1-octanol for *V. parahaemolyticus* (0.3206), and linalool, 1-octanol, and 1-dectanol for *C. albicans* (0.3664, 0.2692, and 0.3158), it has already been reported that they exerted inhibition effects on some microorganism in previous publications (10, 37, 38, 41, 42). This means that they may also contribute to the antibacterial activity of citrus essential oil. Most of the above antibacterial components of component inhibited the growth of microorganisms by damaging cell membranes probably through the massive accumulation of reactive oxygen species (43–45). Although the above-mentioned compounds have been reported to have related antibacterial activity, no article reported which bioactive may play a leading role in inhibiting a certain organism in citrus essential oils. In this paper, it has been concluded that 1-decanol had the major responsibility for the inhibition effects of citrus EOs on *S. aureus*. α-Terpineol and geraniol were the main factors in inhibiting *E. coli* and *C. albicans*. Linalool and geraniol exerted the most anti-*V. parahaemolyticus* effect in citrus EOs.

## Conclusions

Crude EOs were extracted from sweet orange, grapefruit, mandarin, and lemon and successfully separated into fractions using a molecular distillation apparatus. The physicochemical characteristics, cleaning ability, and antimicrobial activity of each EO and fraction were systematically evaluated, showing significant differences in chemical composition, molecular structures, and concentrations of components. Sweet orange and mandarin oils consisted largely of terpenes (>93%), including D-limonene, β-myrcene, and β-phellandrene, whereas grapefruit and lemon oils contained more oxygen-containing components like linalool, geraniol, α-citral, and β-citral (15.25–47.53%). The highest levels of oxygen-containing components were found in Fractions 2 samples, whereas Fraction 1 samples contained more terpenes. The color, density, viscosity, refractive index, optical rotation, and solubility in 95% ethanol of each citrus EO were closely related to the composition and relative concentrations of components. Aroma profiles depended on the contents of geraniol, decanal, 1-decanol, hedycaryol, 1-octanol, octanal, citral, and linalool. In keeping with the principle of “like dissolves like,” citrus EOs showed better cleaning ability than acetone and all tended to dissolve fat-soluble pigments. The key components in the inhibition of *S. aureus, E. coli, C. albicans*, and *V. parahaemolyticus* were 1-Decanol, α-terpineol, geranio, l and linalool, respectively. The findings of this study will help optimize the utilization of citrus peel resources and expand future applications of citrus EOs.

## Data Availability Statement

The original contributions presented in the study are included in the article/supplementary material, further inquiries can be directed to the corresponding author/s.

## Author Contributions

FY and HZ: methodology, investigation, conducting experiments, and writing—original draft. GT: methodology and formal analysis. WR: validation and formal analysis. JL: investigation. HX: formal analysis, supervision, and writing—review and editing. JZ: formal analysis, conceptualization, supervision, writing—review and editing, funding access, and support. All authors contributed to manuscript revision and read and approved the submitted version.

## Conflict of Interest

The authors declare that the research was conducted in the absence of any commercial or financial relationships that could be construed as a potential conflict of interest.

## Publisher's Note

All claims expressed in this article are solely those of the authors and do not necessarily represent those of their affiliated organizations, or those of the publisher, the editors and the reviewers. Any product that may be evaluated in this article, or claim that may be made by its manufacturer, is not guaranteed or endorsed by the publisher.
